# Unlocking microbiology revision: MicrobEscape Room as a playful, team-based active learning activity for undergraduate students

**DOI:** 10.3389/fpubh.2026.1894195

**Published:** 2026-09-16

**Authors:** Isabel Murillo, Emma Steveson, Laura Wright, Lydia Mason

**Affiliations:** School of Biochemistry and Biomedical Sciences, Cellular and Molecular Medicine, University of Bristol, Bristol, United Kingdom

**Keywords:** active learning, educational escape room, game, higher education, microbiology, revision

## Abstract

Active learning approaches are increasingly used to enhance student engagement and support meaningful learning in higher education, particularly in content-heavy disciplines such as microbiology. This study evaluates MicrobEscape Room, a microbiology-focused escape room-based activity designed as a collaborative revision activity for first-year undergraduate students. The game was co-created with undergraduate student partners to ensure alignment with learning objectives, such as teamwork, communication, retrieval of microbiology knowledge, and appropriate difficulty calibration. The activity integrates a story-based approach and problem-based tasks that require teams to solve microbiology-related puzzles across a sequence of escape boxes. The activity was implemented across three academic cohorts (2022–2025), with a total of 118 student questionnaires collected, along with informal oral feedback and observational data. Quantitative outcomes were analysed descriptively using 0–10 rating scales, and qualitative responses were summarised using free-text descriptors. Results indicate consistently high student ratings for organisation, clarity, demonstrator support, and peer interaction. Students reported high levels of sociability and team building, as well as positive engagement with microbiology content. Ratings of enjoyment exceeded perceived educational value, although students consistently identified the activity as both fun and educational. Observations further highlighted a collaborative and interactive learning environment supported by the structured classroom setup. These findings suggest that MicrobEscape Room is a feasible and well-received revision strategy that promotes engagement, collaboration, and active learning behaviours in large undergraduate cohorts. The co-creation approach and alignment of game design with disciplinary learning objectives appear to support student’s reported engagement and perceived value of the activity. While this study focuses on student perceptions, future work should incorporate objective measures of learning outcomes to evaluate the impact of escape room approaches on academic performance.

## 1 Introduction

Students in biomedical and life sciences programmes often encounter complex and challenging disciplinary material. Effective revision is therefore essential, not simply as repetition of previously taught content, but as a crucial phase during which students integrate terminology, concepts, and practical understanding to consolidate their knowledge (1). However, traditional revision methods are often passive and may not foster deep engagement with the content. In contrast, active learning, in which students engage directly by doing and reflecting on their work (2), has been shown to enhance engagement, critical thinking, and knowledge construction. The rationale for adopting active learning approaches in revision is supported by research in STEM education. For instance, students in active learning environments demonstrate higher performance in exams and concept tests, whereas those in conventional lectures exhibit higher failure rates (3). These findings have encouraged a shift amongst educators toward student-centred revision methods incorporating collaboration, feedback, and challenging tasks designed to support meaningful learning in demanding subjects. Further, active learning approaches have been shown to reduce achievement gaps when applied intensively (4). To address the need for engaging revision, educators are increasingly designing activities that promote participation, problem-solving, and reflection. Within this context, gamification, the integration of game elements into educational settings, has emerged as a promising strategy to enhance engagement and motivate student participation in learning activities (5, 6).

Gamification is becoming more common in higher education, with educators observing that game-based methods can help undergraduate students stay engaged and build problem-solving skills (7, 8). Educational games create spaces where students can take action, reflect, and receive instant feedback, helping them learn more deeply (9). Escape room games, in particular, have attracted attention for their immersive and interactive nature. While escape rooms started as live-action puzzles, they are now used in education as an innovative tool to encourage engagement, teamwork, and critical thinking (10). The growing use of escape rooms in teaching different disciplines, such as chemistry, occupational therapy, nursing, medical and veterinary education, shows a shift towards playful, hands-on learning and more authentic educational experiences (11–15). Existing literature on educational escape rooms contains very few demographic analyses, mostly limited to age and educational level (10, 16).

Educational escape rooms are usually group activities with a time limit, where students work together to solve related puzzles and reach a goal (17). These activities help students practise teamwork, problem-solving, and thinking out loud with others (12). An effective escape room design should go beyond simple recall and cover all levels of Bloom’s Taxonomy: remembering, understanding, applying, analysing, evaluating, and creating (18–20). By building puzzles that progress from basic to more complex levels of thinking, escape rooms can be effective revision tools, not just games. Studies show that this approach can boost engagement, teamwork, and understanding of the material, and students tend to view these activities positively (10). Reviews also suggest that escape rooms are being used more as teaching tools that support curriculum goals, motivation, and transferable skills such as collaboration (16, 21). Despite these promising findings, the literature also identifies an emerging need for more rigorous and clearer design guidelines, indicating that the educational use of escape rooms remains a developing field (22).

One of the main challenges in creating educational escape rooms is ensuring alignment between learning objectives and the game (23). Learners are naturally motivated by the goal of “escaping” or solving the problems presented, which may not automatically translate into meaningful engagement with disciplinary content. Research shows that escape rooms should be developed through careful planning, testing, feedback, and improvement (24). This process treats escape rooms as structured teaching tools that must be closely aligned with what students need to learn to achieve the educational goals (25). Frameworks like escapED suggest that educational escape rooms should focus on human interaction, teamwork, and play rather than solely on new technology (26). Overall, the success of escape rooms in education depends on how well the game design supports the intended learning outcomes.

Within this context, “escape boxes” offer a practical and scalable way to bring escape room experiences into the classroom. Escape boxes break down the immersive elements of escape rooms into smaller, flexible parts, making them easier to use when time, space, or resources are limited, while still encouraging teamwork and problem-solving (27). Key design points include ensuring puzzles align with learning objectives, encouraging active team participation, and balancing learner independence with teacher support and guidance so that learner autonomy is supported without excessive cognitive overload (10). These factors are especially relevant in microbiology, where effective revision means not just recalling facts but integrating multiple concepts and applications into problem-based scenarios.

An important extension of this design perspective is the role of students as co-creators in the design and development of educational escape rooms. Students as partners help shape the activity, enabling the incorporation of learner insight into the calibration of difficulty, relevance and engagement (28–30). This aligns with broader co-creation approaches, which emphasise collaboration with learners to improve alignment and innovation, recognising that students possess practical knowledge of their own challenges and learning needs (31). Involving students in creating escape rooms also helps them integrate knowledge and learn from one another, strengthening the link between the game and learning goals (32, 33). This is especially valuable during revision, when the appropriate level of challenge and clarity of progression are essential to keep students motivated and to support their learning.

This study aims to evaluate the perceived learning value of MicrobEscape Room, a microbiology-focused escape room-based activity developed as a revision tool for first-year undergraduate students in a biomedical and life sciences programme. Specifically, the study investigates whether this co-created, narrative-driven activity enhances student engagement and active learning during revision. In the research design, undergraduate students who had recently completed the relevant module collaborated on the activity’s development to ensure that content difficulty and revision needs were authentically calibrated to learner perspectives. This study offers a novel contribution to escape-room pedagogy by documenting a fully co-created activity developed with undergraduate student partners from the outset. The intervention places microbiological concepts within a problem-solving scenario, requiring teams to work collaboratively to solve puzzles directly linked to core curriculum content. This paper contributes to the growing field of escape-room pedagogy in higher education by examining the design and implementation of a student co-created, discipline-specific escape room as a revision strategy. By integrating principles from gamification, design-based research, and collaborative learning, MicrobEscape Room offers a case analysis of how educational escape rooms can be systematically developed to align playful learning with curricular goals in biomedical and life sciences.

## 2 Materials and methods

This study was designed to evaluate student perceptions of MicrobEscape Room, a microbiology-focused escape room-based activity, using a descriptive educational evaluation approach. The activity was developed and implemented at the University of Bristol, and its content was aligned with the first-year microbiology unit Fundamentals of Molecular Microbiology, run in the first teaching block of the academic year. The project was funded by the Faculty of Health and Life Sciences Innovation in Education Funding.

### 2.1 Anatomy of the game

The initial step involved recruiting three students to contribute as co-creators during the summer following their final exams. Eligibility required that each student had completed the relevant unit, ensuring that the knowledge and difficulty were appropriately calibrated, challenging but not overwhelming, and that the scope of unit content for revision was broadened. Student partners are shown in Figure 1.

**Figure 1:**
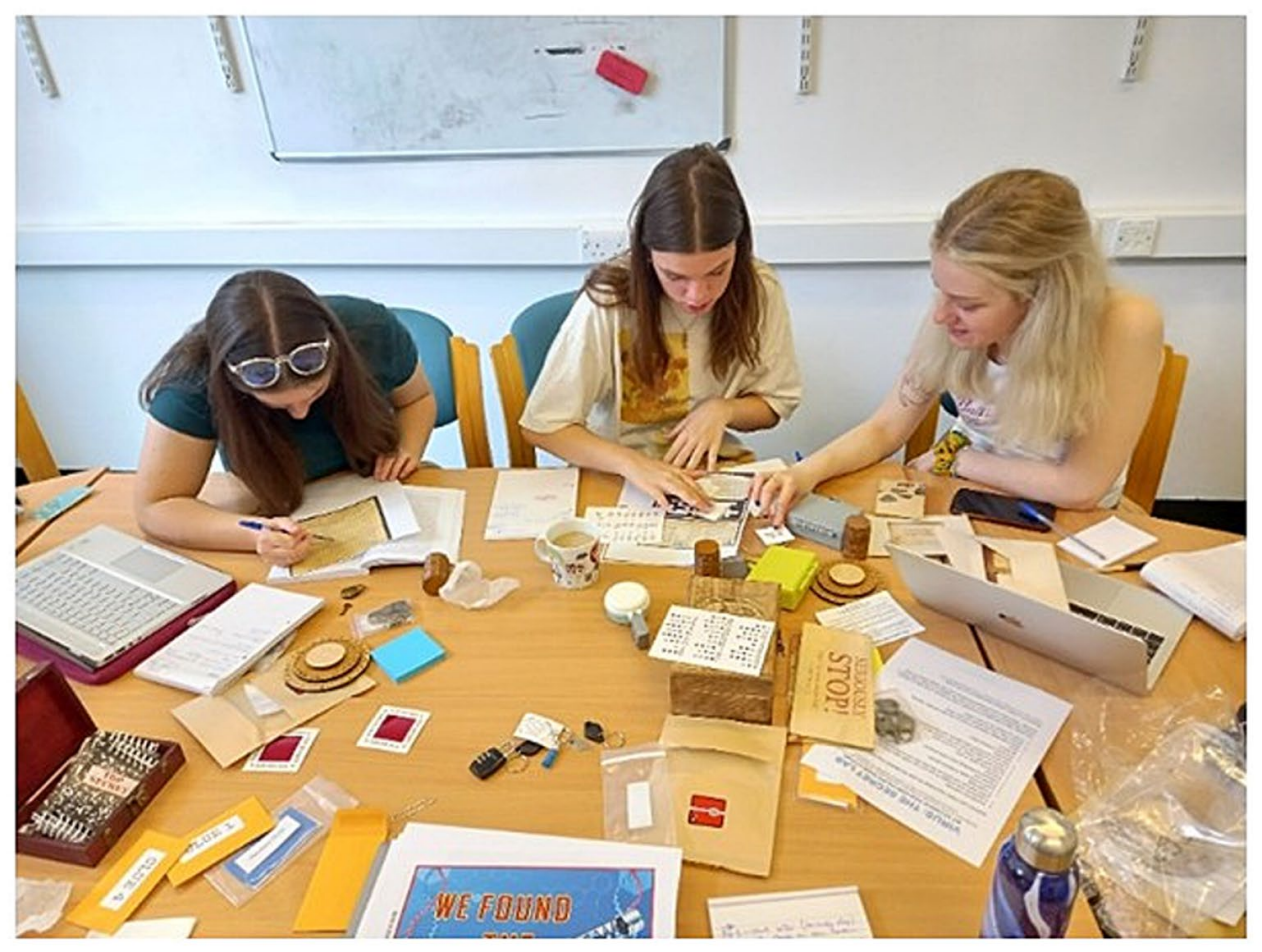
Undergraduate student co-creators working on the design of MicrobEscape Room. The image shows students engaged in the collaborative development of game materials and puzzle design following completion of the microbiology unit, illustrating the co-creation approach adopted in this study. Images are shown with permission of participants.

During game development, relevant literature was reviewed to establish the pedagogical foundation for the activity. Bloom’s taxonomy was used to define the levels of knowledge required for different components of the game. The primary objective was to solve an outbreak caused by a human pathogen, a topic closely aligned with the microbiology unit. The game incorporates props commonly used in laboratory practicals to enhance immersion and establish a microbiology context, such as nitrile gloves, a Petri dish and universal tubes.

Next, the anatomy of the game was defined as escape boxes and envelopes. To promote sustainability, each set is contained within a fabric bag. Once it was decided that three boxes were the optimal number, the puzzles were designed and distributed across the boxes (Figure 2).

**Figure 2:**
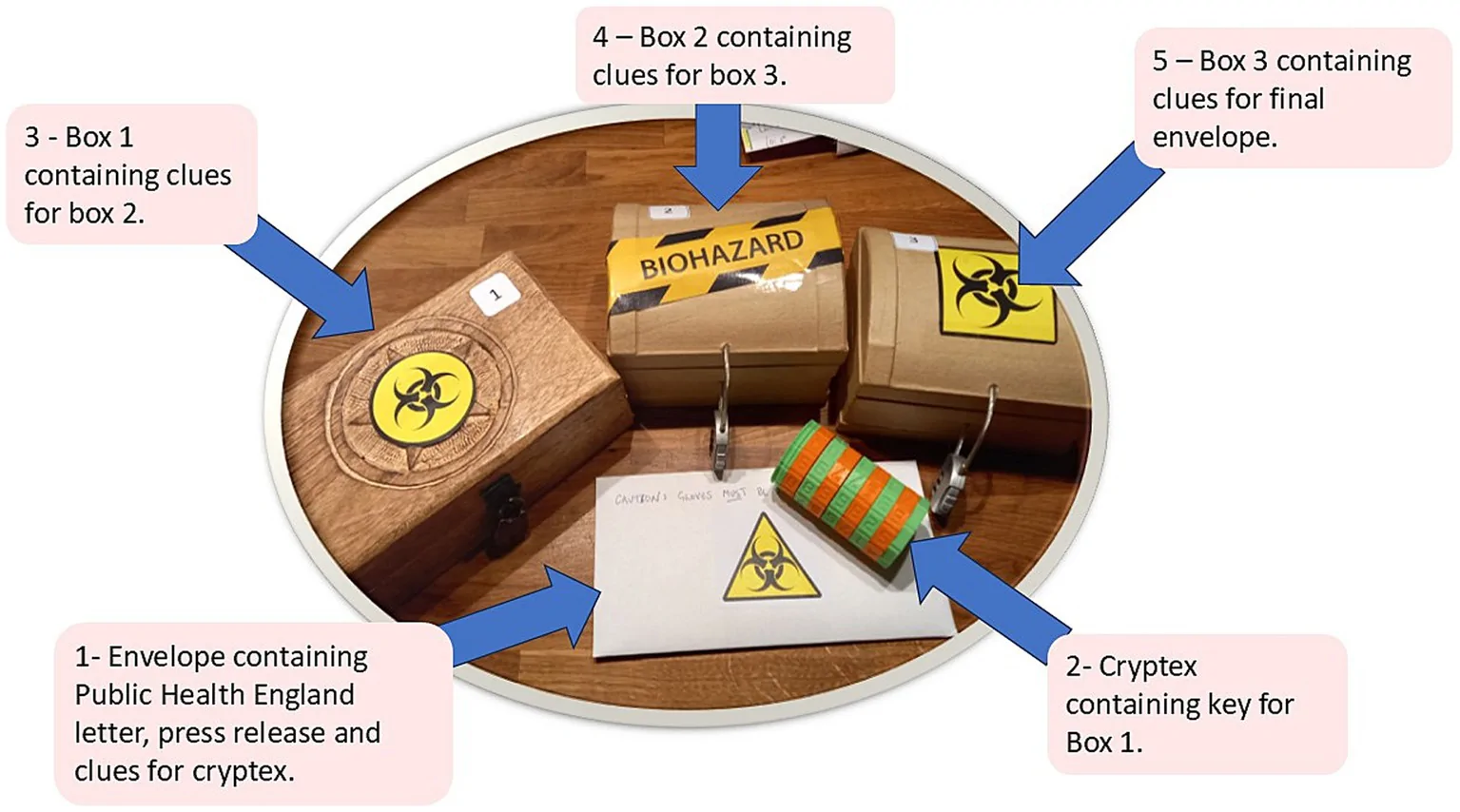
Anatomy of the MicrobEscape Room game. The figure illustrates the structure and progression of the escape-box activity, including the envelope, cryptex, and sequential boxes used to guide gameplay. Arrows indicate the order in which clues are accessed, with each stage designed to support progressive problem-solving and integration of microbiology concepts.

Each set comprises an envelope, a cryptex, and boxes 1, 2, and 3. The contents of each set are depicted in Figure 3:

(i) An envelope containing a letter from the Public Health England, presenting the game challenge and a press release, where the first clue is found. Students need to find the dates of the first vaccine and of the eradication of smallpox. The combination of dates will open a cryptex containing a key that opens the first box.
(ii) Box 1 contains an encrypted message which needs to be deciphered. The message asks a question about microscopy, and the answer provides the code to open the padlock on the second box.
(iii) Box 2 contains a word search on bacterial gene regulation and a set of cards on the virus life cycle and the Baltimore classification. This provides the code for the padlock on the third box.
(iv) Box 3 contains a decoded list, a universal test tube with multiple-choice microbiology questions, and a final envelope with a padlock containing the solution to the original problem, enclosed in a plastic syringe.
(v) There are clues spread across all boxes that provide the code for the last padlock. Every item contributes to the progression of the game and to achieving the solution to the outbreak.

**Figure 3:**
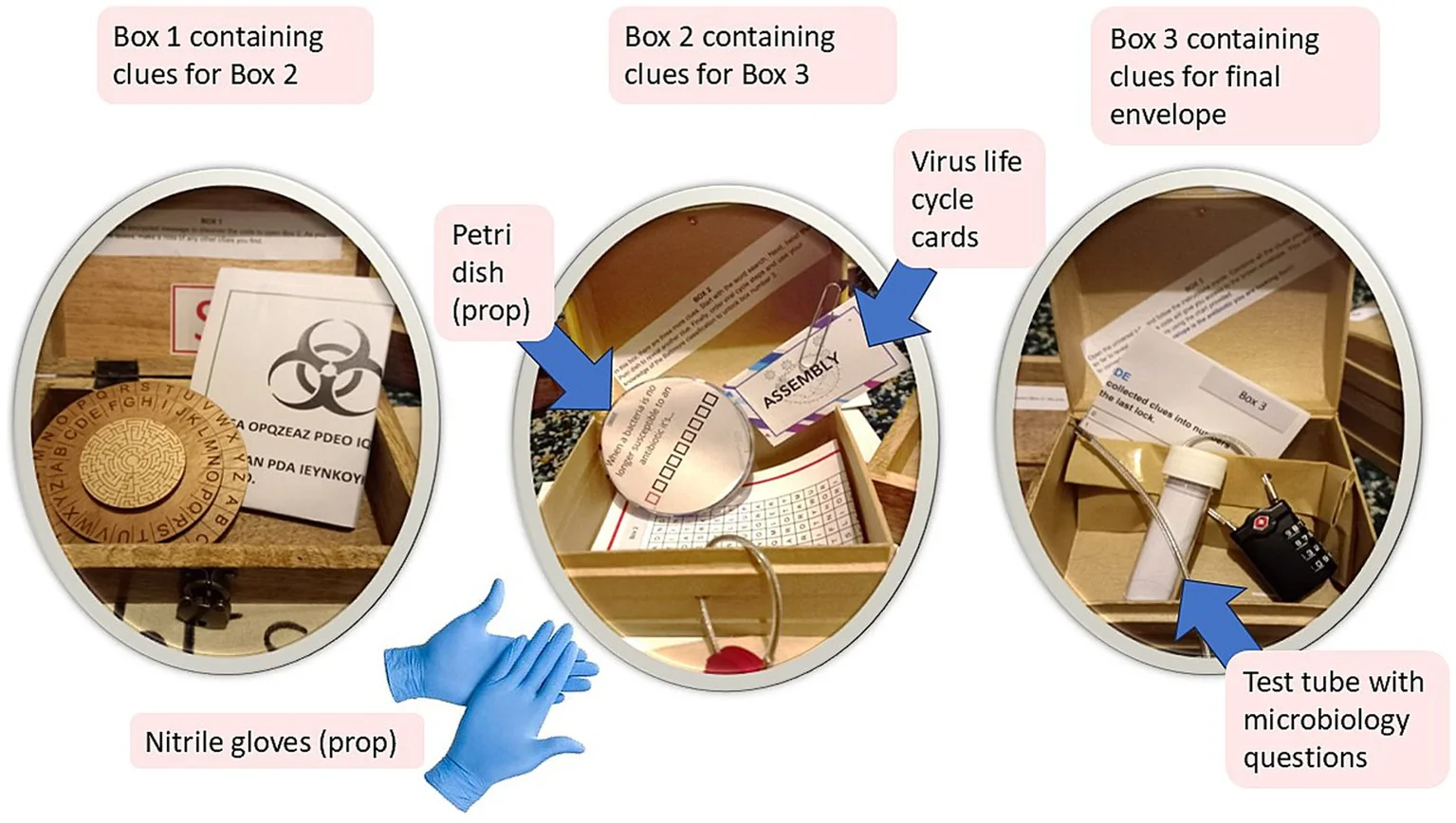
Scientific and laboratory-based components of MicrobEscape Room. The figure shows the contents of Box 1, Box 2, and Box 3, including puzzle elements and microbiology-themed materials used to structure gameplay progression and support disciplinary learning.

Progression from box to box requires students to recall microbiology knowledge taught in the unit, ensuring that each puzzle reinforces key concepts while contributing to the overall solution of the outbreak scenario.

### 2.2 The mechanics of the game

Following a pilot test with a small number of participants, the game was incorporated into the curriculum, supporting an evidence-based approach to adoption, and has been implemented for several consecutive years. Participation in the activity was not mandatory, and students could opt out without academic penalty. The duration of the revision session was originally set at 1 h but was intentionally extended to 1.5 h in response to student feedback indicating that additional time would enhance engagement and activity completion. Scheduling the session during the final week of teaching block 1, immediately before the final unit exam, was a strategic decision to maximise its value as a revision tool.

The choice of a venue capable of accommodating up to 150 students, equipped with tables attached to flat screens, was made to facilitate simultaneous participation by small groups while ensuring that instructions and game materials were clearly accessible to all participants in a large-cohort setting. Allowing students to self-select into groups of five to six and providing each group with a bag containing a game set were key design decisions intended to promote autonomy, collaborative learning, and efficient distribution of tasks. At the start of the session, the game, its mechanics, rules, and hint system are introduced via a PowerPoint presentation to ensure clarity and consistency. Hints are provided at regular intervals throughout the session, a purposeful design element to support equitable progress and maintain engagement across all groups. In line with equality, diversity and inclusion (EDI) considerations, students were also given the option to opt out of group work or to engage with the activity individually if preferred, while still contributing to the teamwork within the session. This approach ensured that participation remained accessible and comfortable for learners with diverse needs and preferences.

### 2.3 Evaluation

The activity formed part of the scheduled teaching for the unit, however, attendance was not mandatory, no register was taken, and students present in the room could choose whether or not to engage with the activity.

At the end of the session, students were invited to complete an anonymous questionnaire to evaluate their experience. The questionnaire was specifically designed to capture student perceptions of the activity and to align with questionnaires used in studies investigating engagement, motivation, and perceived learning. This questionnaire is presented as Supplementary material. In 2025, the questionnaire format was converted to a Microsoft Forms version, enabling digital data collection. All items remained consistent across cohorts except for one question (“educational and fun”), which was converted from a numerical scale to a yes/no format and therefore was not included in the numerical analysis. Questionnaires that were incomplete or not filled in appropriately were withdrawn from analysis to ensure data quality. A total of 118 questionnaires were obtained across three academic years: 35 (2022–23), 51 (2023–24), and 32 (2025–26). Data were analysed separately by cohort and then pooled.

Informal oral feedback was also collected at the end of each session by the academics running the session. Observational notes were made during delivery by members of the academic team. These observations were informal rather than systematic and were used descriptively to contextualise questionnaire findings; therefore, no formal qualitative coding or structured analysis was undertaken.

Questionnaire items were rated on a 0–10 scale and are reported as means (with standard deviations). The number of valid responses varied by item due to missing data. Pooled values were calculated using all available responses across cohorts for each item. Non-numeric entries (e.g., “tick”, “X”) and missing values were excluded from analysis, resulting in item-specific sample sizes (*N*). Standard deviations (SD) were calculated from individual numeric responses using Excel. In addition to means and SDs, the proportion of responses at or above key thresholds (% ≥ 8, high endorsement, and % ≥ 5, moderate endorsement) is also reported to summarise the distribution of responses across items. Percentages were calculated using the item-specific number of valid numeric responses as the denominator. Two questionnaire items used binary (Yes/No) response formats. Descriptive comparisons of questionnaire responses were made across cohorts, focusing on patterns in mean ratings and response distributions.

The evaluation examined student perceptions in several domains, including organisation, clarity of instructions, demonstrator support, peer interaction, perceived difficulty, perceived challenge, perceived revision usefulness, engagement with microbiology, sociality, team building enjoyment and perceived educational value. Aside from the format change to one item in 2025, the questionnaire items and administration procedures were consistent across cohorts, allowing responses to be compared across years.

This study adhered to institutional ethical requirements. Participants provided informed consent for the use of anonymised questionnaire data and for photographed images to be included in publications.

## 3 Results

Results were based on (i) analysis of anonymised questionnaires completed by first-year undergraduate students who participated in the MicrobEscape Room activity across three cohorts (2022: *n* = 35; 2023: *n* = 51; and 2025: *n* = 32), (ii) informal oral feedback collected at the end of sessions, and (iii) observations from the student co-creators and academic supporting delivery. Across the dataset, not all respondents answered every questionnaire item, so the number of valid responses (*N*) varied by question and is reported per item in the tables.

Observational notes indicated a playful and collaborative atmosphere (Figure 4). Following the session briefing, groups began simultaneously and initially attempted early puzzles collectively before distributing tasks within groups as the game progressed. This division of labour appeared to increase efficiency and maintain engagement, consistent with the game’s structure, which requires all components to reach the end goal.

**Figure 4:**
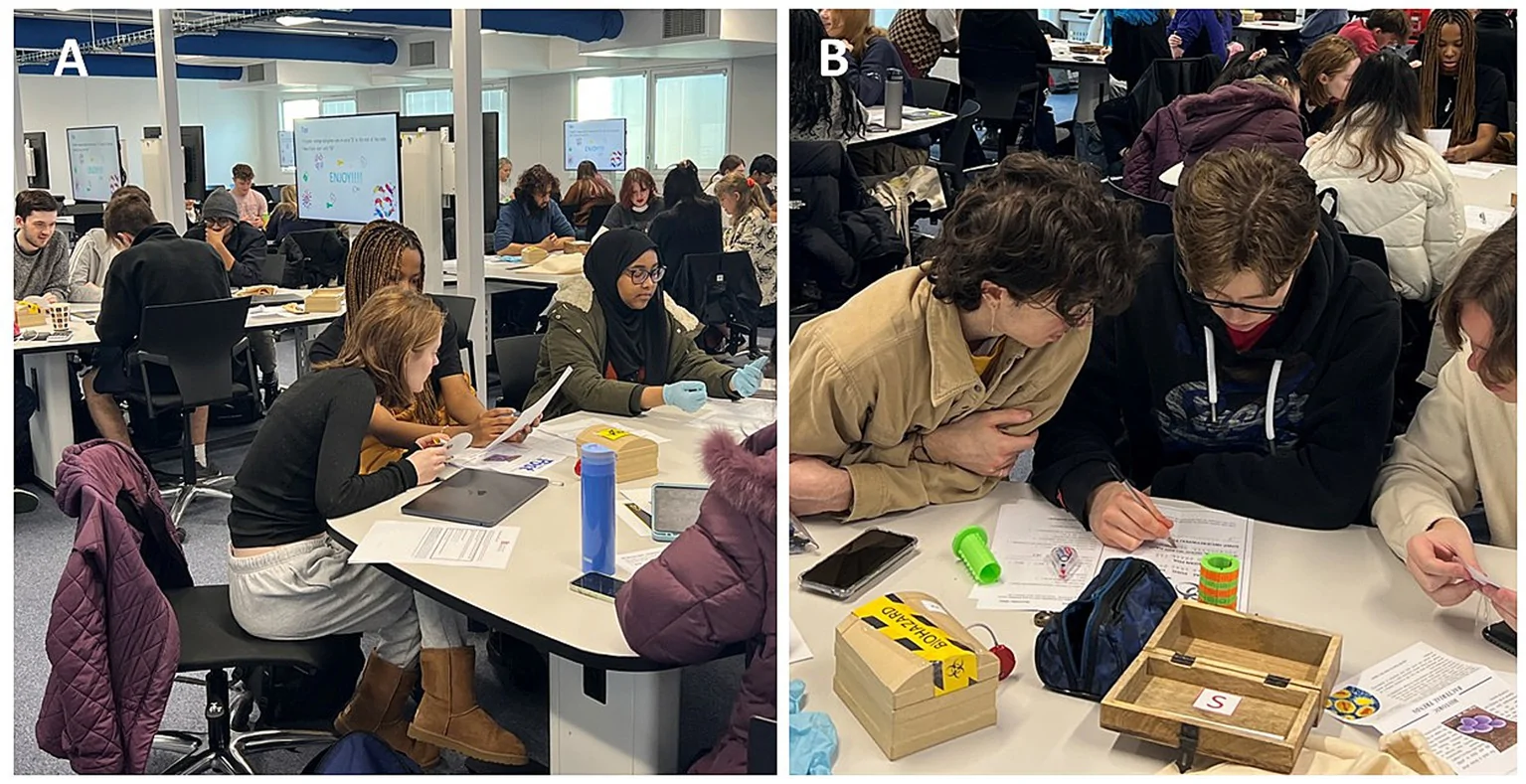
Students participating in the MicrobEscape Room activity during the revision session. **(A,B)** Wider views of the teaching space, showing multiple table-based workstations enabling simultaneous participation across a large cohort. Images are shown with permission of participants.

### 3.1 Pooled questionnaire outcomes (2022–2025 combined)

As shown in Table 1, key quantitative results, pooled across all cohorts, indicate consistently high perceived ratings in several areas central to the session’s experience. Organisation was rated at 9.39 out of 10, clarity of instructions at 9.01, and demonstrator support at 9.47, indicating strong facilitation and structure throughout the activity. Peer interaction metrics were also high, with comfort sharing ideas rated at 9.75 and feeling that opinions were valued at 9.46, reflecting a collaborative and supportive environment. Regarding the activity’s demands, the mean score for perceived difficulty was 8.42, while the reported level of challenge was rated at 7.72, suggesting that students viewed the session as appropriately demanding yet accessible. In terms of learning outcomes, students rated the revision usefulness of the session at 7.62 and reported increased engagement with microbiology at 8.30, indicating that students perceived the session as helpful for revision and recalling microbiology content. Social aspects, such as sociability (9.53) and team building (9.54), were also rated highly, underscoring the importance of collaborative interactions. These quantitative results indicate that students perceived MicrobEscape Room as socially engaging and useful for revision.

**Table 1:** Pooled questionnaire outcomes (2022–2025 combined).

Item	*N*	Mean	SD	% ≥ 8	% ≥ 5
Session well organised	113	9.39	1.04	92.9	100.0
Instructions clear	113	9.01	1.45	85.8	99.1
Demonstrators approachable/helpful	112	9.47	1.21	90.2	99.1
Comfort sharing ideas	112	9.75	0.59	99.1	100.0
Opinion valued by peers	112	9.46	1.15	94.6	99.1
Difficulty adequate	112	8.42	1.92	75.0	96.4
Activity challenging	112	7.72	2.05	56.2	92.0
Revision opportunity	106	7.62	2.18	50.9	94.3
Engagement with microbiology	104	8.30	1.86	69.2	97.1
Sociable activity	105	9.53	0.87	97.1	100.0
Team-building experience	105	9.54	1.35	95.2	100.0
Encourage more alternative activities	73	9.14	1.63	86.3	97.3

% ≥ 8 = (count of numeric responses ≥ 8) ÷ (count of numeric responses) × 100.

% ≥ 5 = (count of numeric responses ≥ 5) ÷ (count of numeric responses) × 100.

Student ratings are reported as mean ± standard deviation (SD) on a 0–10 scale. *N* indicates the number of valid responses for each item. The proportion of responses ≥8 (% ≥ 8) and ≥5 (% ≥ 5) are shown to indicate high and moderate endorsement, respectively.

### 3.2 Educational and enjoyable: “fun” versus “educational”

Across the 2022 and 2023 cohorts combined (paired *N* = 66; 2022 *n* = 22, 2023 *n* = 44), ratings for FUN (mean ± SD: 9.48 ± 0.95) were higher than ratings for EDUCATIONAL value (mean ± SD: 8.00 ± 1.97). In 2025, all respondents described the activity as both educational and fun in the “This activity is both” field (32/32), but this item was recorded as a yes/no response rather than a numerical rating due to the questionnaire being converted from a manual format to an electronic Microsoft Forms version. As a result, no numerical value was collected for this question in 2025 (Table 2).

**Table 2:** Ratings of “educational” and “fun” value (2022–2023 cohorts).

Year	Measure	*N*	Mean ± SD
2022	Educational	22	8.05 ± 1.86
2022	Fun	22	9.82 ± 0.50
2023	Educational	44	7.98 ± 2.04
2023	Fun	44	9.32 ± 1.07

Mean ± SD ratings (0–10 scale) are shown for items assessing perceived educational value and enjoyment. Only respondents with paired ratings for both items were included. The questionnaire changed in 2025, and numerical ratings were not collected.

### 3.3 Session duration changes and responses to “longer session”

The scheduled session length increased from 1 h (2022) to 1.5 h (2023 and 2025), based on the student feedback and demonstrators’ observations during the 2022 activity. More logistical time was needed to set up and finish the game. Students’ responses to “I would have benefited from a longer session” indicated that the increase in session length was a welcome intervention, although a small proportion of students considered that 1.5 h should also be increased (Table 3).

**Table 3:** Responses to “Would have benefited from a longer session” by cohort.

Year (session length)	Yes	No	Do not know	Missing/Other	Total
2022 (1 h)	18	15	0	2	35
2023 (1.5 h)	18	32	0	1	51
2025 (1.5 h)	7	17	7	1	32

Counts of responses (Yes, No, Do not know, Missing/other) are shown for each academic year.

### 3.4 Qualitative descriptors (word cloud)

Students provided brief descriptions of their experiences as a free text. A word cloud summarising these descriptors is shown in Figure 5. In a word cloud, the size of the word indicates the abundance of that word. The word “FUN” is the largest, indicating that it was the most used by students. “ENGAGING” is the second largest. The words “REVISE” and “EDUCATIONAL” are also highlighted.

**Figure 5:**
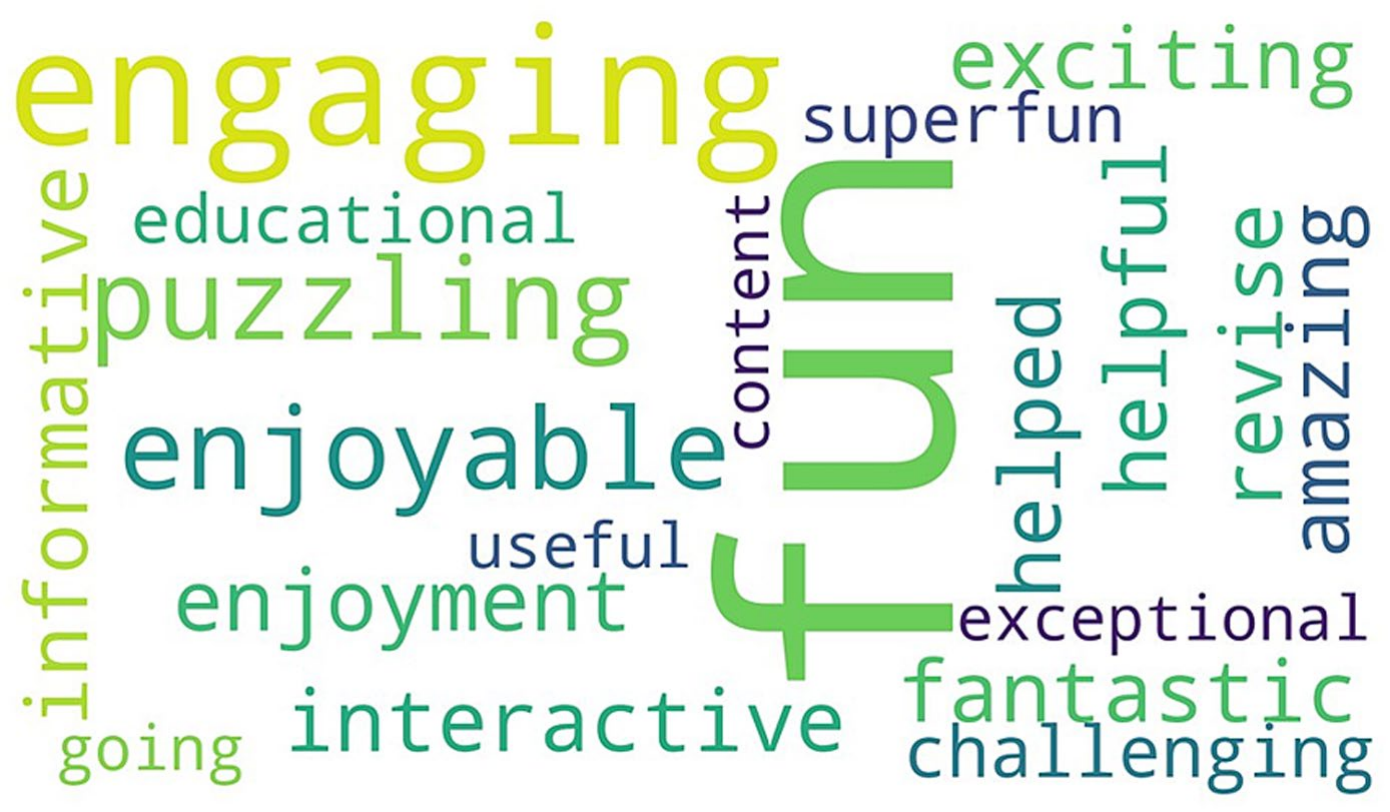
Word cloud of student descriptors of the MicrobEscape Room experience. Word size reflects the frequency of terms used in free-text responses. Common filler words were excluded, and terms represent direct student descriptors of the activity.

## 4 Discussion

MicrobEscape Room progressed from a small-group pilot to a curriculum-embedded workshop and achieved consistently high student ratings across cohorts, supporting feasibility and acceptability as a revision activity. Importantly, this evaluation focused on student perceptions, acceptability, implementation outcomes, and the microbiology knowledge or recall embedded within the game tasks. However, this study did not assess whether participation was associated with improved exam performance or objectively measured learning gains. Therefore, alternative explanations such as self-selection of more motivated students or the influence of novelty effects cannot be ruled out, and the impact of MicrobEscape Room on formal academic outcomes will be explored in the future.

### 4.1 Interpreting the findings through active learning and retrieval practise

The consistently positive ratings for organisation, clarity, support, and peer interaction align with an active-learning perspective, in which students construct understanding through engagement and reflection rather than engaging passively (2, 3). This is further supported by constructivist learning theory, which emphasises that students learn most effectively when they actively construct knowledge through experience and social interaction. Specific MicrobEscape Room elements put these theories into practise: for instance, collaborative puzzle-solving mirrors social constructivism by requiring students to negotiate meaning and share reasoning within teams (34).

The sequential puzzle pathway also provides rapid performance information as teams progress, consistent with formative feedback processes (35, 36). Moreover, tasks such as recalling microbiology content to unlock codes and analysing clues to solve the case embed guided retrieval practise, consistent with retrieval-based learning principles (1). Although learning gains were not assessed, student perceptions of engagement and revision usefulness suggest that the activity was experienced as supportive within their revision process. As the evaluation relied on self-reported perceptions, conclusions relate to perceived educational value rather than demonstrated learning outcomes. Students rated the activity as enjoyable, with “fun” scores higher than “educational” scores, across both scales. From a motivational perspective, this pattern is consistent with the role of game elements in shaping learning-relevant behaviours, such as persistence and effort, through gamified elements (37). In high-content subjects such as microbiology, affect and motivation may be particularly important during pre-exam revision periods, when anxiety and perceived workload can otherwise push students toward low-effort strategies.

### 4.2 Cohort consistency

Within cohorts and across years, responses to the 0–10 scale questions were broadly similar between years, with more variation within each year than between years. Regarding session length, fewer students felt that additional time would be beneficial even after the duration was increased. Overall, these findings suggest a broadly stable student experience across years, and this consistency across cohorts is beneficial, as supportive learning environments are associated with improved student engagement and confidence (38, 39).

### 4.3 Constructive alignment and cognitive demand

A key pedagogical challenge in educational escape rooms is ensuring that gameplay remains aligned to disciplinary learning outcomes rather than becoming an “activity” disconnected from content (23). The design of MicrobEscape Room, which links each puzzle to unit concepts and requires teams to integrate clues across components and recall knowledge from the taught material, reflects constructive alignment in practise, with the activity structure, disciplinary content, and assessment signals coherently mapped to support perceived learning values. In addition, escape-room tasks can be deliberately pitched across cognitive levels (18), enabling revision to extend beyond recall into application, analysis and evaluation. The observed pattern where “difficulty adequate” ratings exceeded “challenging” ratings suggests that the activity was appropriately demanding while remaining accessible across teams; such calibration is consistent with guidance in escape room design frameworks that emphasise playtesting, pacing and scaffolding (10, 24).

### 4.4 Positioning within escape-room pedagogy and design frameworks

Recent reviews characterise educational escape rooms as collaborative, time-bounded designs that can support motivation, curriculum engagement and transferable skills, while also highlighting the need for more transparent and rigorous evaluation approaches (16, 22). Our multi-cohort findings contribute implementation evidence for acceptability and perceived value in a large-cohort undergraduate setting. Importantly, scalability is a recurrent implementation challenge in the literature; the “escape box” approach offers a pragmatic classroom adaptation that can preserve collaboration while easing constraints of space and staffing (27). MicrobEscape Room follows this escape-box model, enabling consistent classroom delivery and sustained use after initial piloting. Design frameworks such as EscapED and Room2Educ8 emphasise a structured process including narrative, puzzle sequencing, briefing/debriefing, prototyping and iterative refinement (10, 26). The project’s year-on-year adjustments (including extending the session duration and refining information provided to support early puzzle progress) are consistent with this iterative, design-based approach.

### 4.5 First-year student cohorts

First-year curricula are widely argued to require intentional design that supports students’ academic transition while also creating an involving and social learning environment (40, 41). Similarly, evidence from the “What Works?” programme in the UK identifies students’ sense of belonging, built through peer relations, meaningful staff interaction and an explicit academic purpose, as a critical contributor to retention and success (42). In this context, MicrobEscape Room’s strongest outcomes were social (sociability and team building) alongside high ratings for psychological safety (comfort sharing ideas and feeling valued). These characteristics map directly onto “transition pedagogy” principles and a belonging-oriented view of the first-year experience, suggesting that the intervention may serve dual purposes: structured revision and cohort-building. This interactive activity provides students with social opportunities to meet new peers and supports the development of a sense of belonging, a factor identified as critical for student engagement, retention, and success in higher education (42).

### 4.6 Inclusivity

Some students preferred to work individually. The game, rules, and number of puzzles were designed to provide students with flexibility, so they could play collaboratively or individually within their groups. As all the pieces were relevant to the game’s goal, students working individually also contributed to their group’s results, enhancing a sense of teamwork. The venue used for the activity was chosen to accommodate accessibility needs. This interactive activity provides an inclusive learning environment in which students can participate in different ways, supported by peers and academic staff, which contributes to the development of a sense of belonging (42, 43).

### 4.7 Co-creation as a design strategy

The development model involving undergraduate co-creators aligns with the increasing emphasis on student-staff partnership in learning and teaching, where students contribute insights into relevance, pacing, and clarity (28, 29, 44). In the context of escape rooms, co-creation may be particularly advantageous, as it allows activities to be shaped by students’ perspectives, helping to ensure appropriate levels of challenge and clarity in puzzle design. Additionally, such student–staff collaboration provides opportunities for students to develop transferable and professional skills, including communication, responsibility, and collaboration, which are associated with their future academic and professional development (45, 46). Existing publications typically describe staff-designed escape rooms, with students involved mainly in piloting or evaluation. The present study therefore extends this work by demonstrating how co-creation can be embedded within the design of a discipline-specific escape-room intervention.

### 4.8 Limitations and future directions

This study has some limitations that could be addressed in the future. As with many curriculum-embedded educational innovation studies, this evaluation was conducted within a single institution and relied on students’ participation and self-reported perceptions, which may affect applicability across institutions. However, collecting data from three different cohorts enhanced consistency. The study did not include objective measures of learning or retention. Positive perceptions may partly reflect the novelty and playful nature of the format, and such enthusiasm does not necessarily correspond to learning gains. This distinction should be considered when interpreting the findings.

Educational escape room studies in the health sciences have demonstrated that learning outcomes can be assessed directly; for instance, a peer-to-peer physiology escape room showed that participants achieved higher exam scores than non-participants and received positive qualitative feedback (47). Building on these findings, future research on MicrobEscape Room should move beyond student perceptions to incorporate more objective measures of educational impact. For example, implementing low burden pre- and post-quizzes aligned with the puzzle content could provide objective evidence of knowledge acquisition attributable to the intervention. Where feasible, comparing exam-item performance between participants and non-participants would further clarify the relationship between escape-room engagement and academic outcomes. Furthermore, qualitative methods such as reflective interviews could shed light on how playful, team-based activities influence revision strategies, motivation, and retention. Expanding the evaluation in these ways would offer a deeper understanding of the effectiveness of playful learning interventions in supporting both engagement and meaningful learning gains during revision.

## 5 Conclusion

The evaluation of this activity shows that students perceived the co-created MicrobEscape Room activity as engaging, collaborative and useful for revision. Although the study did not assessed objective learning outcomes, the consistently positive perceptions across cohorts indicate that the activity was well received and may support student motivation and engagement with the unit microbiology content. Future research should incorporate objective measures of learning and retention to determine the educational impact of escape-room activity approaches in the higher education settings.

## Data Availability

The raw data supporting the conclusions of this article will be made available by the authors, without undue reservation.
