# Supplementary material for: Unlocking microbiology revision: MicrobEscape Room as a playful, team-based active learning activity for undergraduate students
Source: Front Public Health. 2026 Sep 16;14:1894195. doi: 10.3389/fpubh.2026.1894195 (PMC13624095; doi:10.3389/fpubh.2026.1894195)
Supplement: Supplementary file 1 [file Data_Sheet_1.docx]

Supplementary Material

# 1 Supplementary Data

Supplementary Material should be uploaded separately on submission. Please include any supplementary data, figures and/or tables.

Supplementary material is not typeset so please ensure that all information is clearly presented, the appropriate caption is included in the file and not in the manuscript, and that the style conforms to the rest of the article.

# 2 Supplementary Figures and Tables

For more information on Supplementary Material and for details on the different file types accepted, please see [here](https://www.frontiersin.org/guidelines/author-guidelines#supplementary-material).

## 2.1 Supplementary Figures

**Supplementary Figure 1.** Questionnaire used during the MicrobEscape Room activity to collect student feedback on enjoyment, perceived educational value, and overall experience.
